# Insulin resistance and triglyceride/HDL_c _index are associated with coronary artery disease

**DOI:** 10.1186/1758-5996-2-11

**Published:** 2010-02-03

**Authors:** Marcello C Bertoluci, Alexandre S Quadros, Rogério Sarmento-Leite, Beatriz D Schaan

**Affiliations:** 1 Internal Medicine Department, Hospital de Clínicas de Porto Alegre, Universidade Federal do Rio Grande do Sul, Brazil; 2 Institute of Cardiology of Rio Grande do Sul/University Foundation of Cardiology, Porto Alegre, Brazil; 3 Endocrine Division, Hospital de Clínicas de Porto Alegre, Universidade Federal do Rio Grande do Sul, Brazil

## Abstract

**Background:**

Insulin-resistance is associated with cardiovascular disease but it is not used as a marker for disease in clinical practice.

**Aims:**

To study the association between the homeostatic model assessment (HOMA-IR) and triglyceride/HDLc ratio (TG/HDLc) with the presence of coronary artery disease in patients submitted to cardiac catheterization.

**Methods:**

In a cross-sectional study, 131 patients (57.0 ± 10 years-old, 51.5% men) underwent clinical, laboratory and angiographic evaluation and were classified as No CAD (absence of coronary artery disease) or CAD (stenosis of more than 30% in at least one major coronary artery).

**Results:**

Prevalence of coronary artery disease was 56.7%. HOMA-IR and TG/HDLc index were higher in the CAD *vs *No CAD group, respectively: HOMA-IR: 3.19 (1.70-5.62) vs. 2.33 (1.44-4.06), p = 0.015 and TG/HDLc: 3.20 (2.38-5.59) vs. 2.80 (1.98-4.59) p = 0.045) - median (p25-75). After a ROC curve analysis, cut-off values were selected based on the best positive predictive value for each variable: HOMA-IR = 6.0, TG/HDLc = 8.5 and [HOMA-IR×TG/HDLc] = 28. Positive predictive value for coronary artery disease for HOMA-IR>6.0 was 82.6%, for TG/HDLc>8.5 was 85.7% and for [HOMA-IR×TG/HDLc]>28 was 88.0%. Adjusted relative risk (age, gender, diabetes, body mass index, systolic blood pressure) for the presence of coronary artery disease was: for HOMA-IR>6.0, 1.47 (95.CI: 1.06-2.04, p = 0.027), for TG/HDLc>8.5, 1.46 (95% CI:1.07-1.98), p = 0.015) and for [HOMA-IR × TG/HDLc] >28, 1.64 (95%CI: 1.28-2.09), p < 0.001).

**Conclusions:**

Increased HOMA-IR, TG/HDLc and their product are positively associated with angiographic coronary artery disease, and may be useful for risk stratification as a high-specificity test for coronary artery disease.

## Background

Insulin resistance is associated with cardiovascular disease [1]. Possible mechanisms include induction of pro-inflammatory and pro-coagulant states which are detrimental to endothelial function and may play an important role in mediating atherogenesis [2].

Homeostatic model assessment of insulin resistance (HOMA-IR) has emerged as a practical and simple method for estimating insulin resistance. This index was extensively validated in comparison with the gold-standard method for the evaluation of insulin resistance, the hyperinsulinemic euglycemic glucose clamp technique [3]. While this method is currently not routinely used as a cardiovascular risk marker, we hypothesized that it could be potentially useful since hyperglycemia and hyperinsulinemia are both related to cardiovascular disease. Also, since triglycerides and HDL are independent predictors of cardiovascular risk [4], their ratio could be used as a simple cardiovascular risk marker.

The aim of the present study was to study the association between HOMA-IR and the triglyceride/HDLc ratio with the presence of coronary artery disease in patients who were submitted to coronary angiography and to define the best cut-off values for clinical practical use.

## Methods

This was a cross-sectional study with patients referred for coronary angiography at a reference center for interventional cardiology. Between December 2002 and December 2003, 150 patients were screened and 131, who presented inclusion criteria and accepted to participate, were included. Inclusion criteria were age between 30 and 80 years old, chest pain, documented ischemia or clinical referral for coronary angiography by the attending physician. Exclusion criteria were: previous myocardial infarction in the last 60 days, heart transplantation or revascularization procedure, known malignant neoplasia, haemodialysis or current insulin therapy.

A clinical questionnaire, physical examination and a 12 h-fasting blood sample collection were performed on all patients immediately before coronary angiography. Each subject gave written consent and the study protocol was approved by the Hospital Ethics Committee.

### Biochemical investigation

On the day of the procedure, a fasting blood sample was obtained for glucose, total cholesterol, high-density cholesterol (HDL-c), triglycerides (TG), creatinine, fibrinogen and ultrasensitive C-reactive protein (CRP). Laboratory measurements were performed using automated enzymatic commercial kits (Roche, Mannheim, GE). Fibrinogen was evaluated on a Fibrintimer II (Dade Behring Inc, Newark, Marburg, Germany) and processed in the auto-analyser (CA-540, Sysmex). The concentration of low-density cholesterol (LDL-c) was calculated with Friedwald's formula. Serum insulin was determined by enzyme immunoassay commercial kits (Abbott-Murex, Park, IL, USA) and CRP by nephelometry (nephelometer BN100, Dade Behring Inc., Marburg, Germany). Insulin resistance was assessed by the HOMA-IR (homeostasis model assessment of insulin resistance) method, as previously described [5].

### Coronary angiography

Coronary angiographies were performed using Siemens Axiom Artis equipment (Munich, Germany), and all procedures followed standard routines. The coronary angiograms were evaluated by two experienced interventional cardiologists, unaware of the patients' clinical presentation. Lesions quantification were performed using a quantitative coronary angiography (QCA) software and were stratified in mild (up 30% in lumen narrowing), moderate (30 to 70% in lumen narrowing) or severe (greater than 70% in lumen narrowing) disease. Angiographic evidence of coronary artery disease was classified as: No CAD (complete absence of detectable coronary artery disease) or CAD (30% or more stenosis in at least one major coronary artery) to assure coronary artery disease presence and avoid inclusion or misinterpretation of patients with incipient or very mild disease.

### Statistical analysis

Continuous parametric variables such as age, body mass index, systolic and diastolic blood pressure, LDLc, fibrinogen, HDLc, fasting plasma glucose and triglycerides/HDLc ratio were analyzed using the Student *t-test*. Non-parametrical variables, such as TG, TG/HDLc and HOMA-IR were compared between groups by the Mann-Whitney rank test and expressed as median (p25-75). Categorical variables were compared by the *Chi square *test. HOMA-IR was calculated using the following formula [5]:

In a ROC curve analysis, the best positive predictive values for HOMA-IR, TG/HDLc and their product (HOMA-IR×TG/HDLc) were defined as cut-off values for the study. Relative risks were determined between the presence or absence of coronary artery disease (outcomes) *vs *A. HOMA-IR higher/equal vs. lower than the cut-off level of 6.0, B. TG/HDLc index higher/equal vs. lower than the cut-off level of 8.5 and C. The product of HOMA-IR × TG/HDLc higher/equal vs. lower than the cut-off level of 28 interval, using multivariate Poisson regression adjusted for age, sex diabetes, body mass index and systolic blood pressure. For all tests a *P *value ≤ 0.05 was considered to be statistically significant.

## Results

Clinical, biochemical and image data were available for all 131 patients. The mean age of the whole group was 57.8 ± 10.5 years and 51.5% were men. Clinical and laboratory data are shown in Table 1. The percentage of patients with coronary artery disease was 56.5% (CAD group). In the CAD group patients were older, more frequently male and there were slightly more cases of diabetes mellitus. However, body mass index was lower in the CAD group, as compared to those without coronary artery disease. No differences were found regarding the frequency of arterial hypertension, smoking or dyslipidemia.

**Table 1:** Characteristics of the patients

Characteristics N = 131	No CAD N = 57	CAD N = 74	p
Age (years)	55.3 ± 10.0	60.3 ± 10.5	0.006
Male (%)	20 (33.9)	49 (64.5)	< 0.001
BMI (kg/m^2^)	29.8 ± 5.5	27.0 ± 4.4	0.002
Systolic BP (mmHg)	140.4 ± 26.6	140.1 ± 22.4	0.950
Diastolic BP (mmHg)	86.3 ± 17.2	84.3 ± 12.1	0.450
Diabetes Mellitus (%)	15 (25.8)	36 (48.6)	0.005
Arterial Hypertension (%)	40 (68.9)	52 (72.2)	0.650
Smokers (%)	12 (20.3)	25 (34.7)	0.069
Statin users (%)	9 ( 15.2)	15 ( 19.7)	0.499
Oral anti-diabetic agents (%)	5 (8.8)	9 (12.1)	0.482
Physically active (% of total)	12 (20.6 )	25 ( 32.9 )	0.114
HbA1c (%)	5.9 ± 0.9	6.2 ± 1.1	0.180
Fasting plasma glucose (mg/dl)	101.1 ± 33.3	117.7 ± 48.7	0.027
LDLc (mg/dl)	117.2 ± 33.1	121.1 ± 37.3	0.064
HDLc (mg/dl)	49.8 ± 11.7	45.7 ± 10.5	0.036
Triglycerides (mg/dl)	143.0 (95.0-208.0)	170.0 (106.7-219.5)	0.134
C-Reactive Protein (mg/dl)	3.04 ± 2.05	3.24 ± 2.25	0.636
Serum creatinine (mg/dl)	0.96 ± 0.22	1.08 ± 0.35	0.029
Fibrinogen (mg/dl)	336.9 ± 81.2	357.6 ± 97.3	0.488

Data expressed as mean and standard deviation or n (%), except for triglycerides, which is presented as median and p25-p75 range. CAD = coronary artery disease group, No CAD = absence of coronary disease group, BMI = body mass index; BP = Blood pressure; LDL-c = low-density lipoprotein cholesterol; HDLc = high-density lipoprotein; HbA1c = Glycated hemoglobin. Physically active individuals were defined as those who performed at least 150 min of moderate/intense exercise per week.

**Figure 1:**
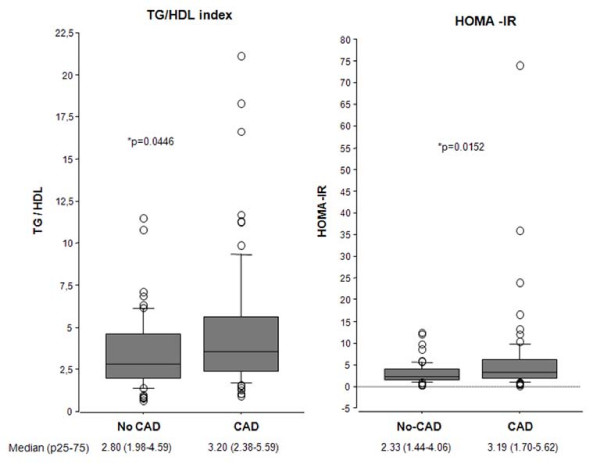
**Triglycerides/HDLc ratio (A) and HOMA-IR (B) in patients with any coronary lesion (CAD, n = 74) or without coronary artery disease (No CAD n = 57)**. Data are median and p25-p75. *P < 0.005.

TG/HDLc index and HOMA-IR were significantly increased in the CAD group (Figure 1), as compared to the No CAD group, (p = 0.0446 and p = 0.0152, respectively). After a ROC curve analysis, the highest positive predictive values were used as cut-off values. The cut-off value for HOMA-IR was 6.0, for TG/HDLc index was 8.5 and for the product [HOMA-IR × TG/HDLc] was 28.0. The positive predictive value for the presence of coronary artery disease with a HOMA-IR >6.0 was 82.6%, for a TG/HDLc > 8.5 was 85.7% and for [HOMA-IR×TG/HDLc]>28 was 88.0% (Table 2).

**Table 2:** Cut-off analysis

Cut-off	Sensitivity (%)	Specificity (%)	PPV (%)
HOMA-IR			
2.0	71.6	45.6	63.1
3.0	56.7	59.6	64.6
4.0	45.9	73.7	69.4
5.0	32.4	84.2	72.7
5.5	29.7	89.5	78.6
6.0	25.7	92.9	82.6
7.0	20.2	92.9	78.9
TG/HDLc			
2.0	85.1	24.1	58.8
3.0	59.4	53.4	62.0
4.0	47.3	68.9	66.0
5.0	32.4	81.0	68.6
6.0	20.2	86.2	65.2
7.0	16.2	93.0	75.0
8.0	16.2	94.8	80.0
8,5	16.2	96.5	85.7
HOMA-IR × TG/HDLc			
4 (p25)	80.8	29.8	59.6
10 (p50)	57.5	59.6	64.6
24 (p75)	34.2	85.9	75.7
28 (p80)	30.1	94.7	88.0

Sensitivity, specificity, positive predictive value (PPV), for different cut-off values of homeostatic model assessment (HOMA-IR), Triglyceride/HDLc index (TG/HDLc) and the product [HOMA-IR×TG/HDLc].

In a multivariate regression, we determined the relative risk for significant coronary artery disease (adjusted for age, gender, body mass index, diabetes and systolic blood pressure) for the variables HOMA-IR above 6.0, TG/HDLc above 8.5 and [HOMA-IR×TG/HDLc] above 28. All variables were associated to significantly increased relative risk for coronary artery disease (Table 3, model A). In order to remove an eventual effect of oral anti-diabetic agents in HOMA-IR, data were re-analyzed without the patients on these medications (n = 14) and relative risks remained significantly increased (Table 3, model B).

**Table 3:** Multivariate regression analysis and relative risks for coronary artery disease for HOMA-IR > 6, TG/HDLc index > 8.5 and [HOMA-IR × TG/HDLc] > 28 with adjustments.

Model A	RR	95% CI	p	adjustments
HOMA IR >6.0	1.178	1.043-1.330	0.008	age, BMI, gender, diabetes, SBP
TG/HDLc >8.5	1.175	1.040.1.330	0.043	age, BMI, diabetes, SBP, gender
[HOMA-IR×TG/HDLc]>28	1.220	1.125-1347	<0.001	age, BMI, diabetes, SBP, gender

**Model B**	**RR**	**95% CI**	**p**	**adjustments**

HOMA IR >6.0	1.161	1.016-1.326	0.028	age, BMI, diabetes, SBP
TG/HDLc >8.5	1.158	1.005-1.335	0.043	age, BMI, diabetes, SBP, gender
[HOMA-IR×TG/HDLc]>28	1.231	1.125-1347	< 0.001	age, BMI, diabetes, SBP, gender

Model A refers to all patients of the study, n = 131. Model B refers to patients without oral anti-diabetic agents (n = 117).

BMI = Body mass index. SBP = Systolic blood pressure. RR = Relative risk. P value was obtained after adjustments.

## Discussion

In the present study, we showed an association between insulin resistance, with the presence of coronary artery disease in patients referred for coronary angiography. A HOMA-IR higher than 6.0 and the TG/HDLc index above 8.5 were associated with the presence of coronary lesions, and it was independent of age, gender, body mass index, diabetes mellitus and systolic blood pressure. This effect was unlikely to be due to the use of anti-diabetic oral agents on HOMA-IR because patients on these medications were removed from the analysis and the results did not change. The product of HOMA-IR and TG/HDLc index had an even stronger association with the presence of coronary artery disease than each variable alone. Considering that these patients were in intermediate-high risk for coronary artery disease, these tests presented a very high positive predictive value.

Insulin resistance evaluated by the HOMA-IR and triglyceride measurements is usually not included in the recommendations for cardiovascular risk stratification [6]. These conditions are generally considered emerging risk factors [7], although two meta-analyses have shown predictive value for cardiovascular outcomes [8,9]. However, we did not find studies using both HOMA-IR and TG/HDLc, as a risk factor.

Although cause-effect conclusions cannot be drawn from cross-sectional studies, the associations observed in the present study raises the hypothesis that these measurements, used together, may optimize stratification of coronary artery disease risk. This is in accordance with prospective studies showing that insulin resistance, as evaluated by HOMA-IR [10] or by the euglycemic hyperinsulinemic clamp [11], indicates that it is associated with the occurrence of major cardiovascular events and coronary heart disease. In one study, increased HOMA-IR was associated with an increased incidence of myocardial infarction and death, even when the cardiovascular risk was adjusted for smoking and low physical activity. Interestingly, in that study, insulin resistance was defined in those subjects who present HOMA-IR in the 75^th ^percentile (1.80 for women and 2.12 for men), a much lower threshold than used in the present study [12].

Hyperinsulinemia was shown to be an independent predictor of complex atherosclerotic lesions detected by echocardiography in the thoracic aorta in non-diabetic patients [13]. Previous studies have investigated the association between angiographically proven coronary atherosclerosis and insulin resistance evaluated by a HOMA-IR higher than 1.8 (the median value). Patients with higher levels of insulin resistance had a more severe, extensive, and distal type of coronary artery disease [14].

Increased TG/HDLc index was also previously shown to be associated with ischemic heart disease. In a previous cohort study, asymptomatic men initially free from ischemic heart disease who presented eletrocardiographic changes after 8 years, had more fatal acute myocardial infarction when increased TG/HDLc was also present, indicating a synergistic effect of both low HDLc and high triglyceride levels [15].

A potential limitation of the present study is that sub-clinical or mild disease could be better assessed using intravascular ultrasound or optical coherence tomography. Also, LDL cholesterol levels, used in most studies and ascertained as classic risk factor for coronary artery disease, are actually estimations calculated with the same variables as in the TG/HDLc relationship with the exception of total cholesterol. Thus, considerable correlation between both values is expected. However, a comparison of TG/HDLc with LDL cholesterol levels is beyond the scope of the present study.

We believe that this simple and non-invasive set of tests may be a useful tool in optimizing the selection of patients to proceed to a more invasive investigation. Further longitudinal studies on insulin resistance and coronary artery disease should be undertaken in order to confirm these data in high-risk patients.

## Conclusions

We conclude that because increased HOMA-IR, TG/HDLc and their product are positively associated with the angiographic coronary artery disease, they may be useful for cardiovascular risk stratification.

## Competing interests

The authors declare that they have no competing interests.

## Authors' contributions

The authors MCB, ASQ, RSL and BDS carried out data collection. MCB performed the statistical analyses. MCB, ASQ, RSL and BDS performed the discussion of results. MCB and BDS performed the study design and alignment and drafted the manuscript. All authors read and approved the final manuscript.
